# Manual rolling load and low back pain among workers in Japan: a cross-sectional study

**DOI:** 10.1093/joccuh/uiae015

**Published:** 2024-04-11

**Authors:** Kazuyuki Iwakiri, Takeshi Sasaki, Tanghuizi Du, Keiichi Miki, Fuyuki Oyama

**Affiliations:** National Institute of Occupational Safety and Health, Kawasaki, 214-8585, Japan; National Institute of Occupational Safety and Health, Kawasaki, 214-8585, Japan; National Institute of Occupational Safety and Health, Kawasaki, 214-8585, Japan; National Institute of Occupational Safety and Health, Kawasaki, 214-8585, Japan; National Institute of Occupational Safety and Health, Kawasaki, 214-8585, Japan

**Keywords:** low back pain, rolling, manual handling, weight, workplace

## Abstract

**Objectives:**

Manual rolling of heavy objects remains in the workplace. The Health and Safety Executive (HSE) in the United Kingdom recommends load weights of <400 kg in the rolling task. However, the association of rolling weights <400 kg with work-related low back pain (LBP) has not been sufficiently investigated. This study examined the effect of rolling loads weighing <400 kg on LBP among Japanese workers.

**Methods:**

A web-based survey gathered information from 15 158 workers in 2022. Among them, 15 035 did not handle loads, whereas 123 handled rolling weights <400 kg. Load weight was categorized into 4 groups: no-handling (0 kg) and rolling weights of ≤20, 20-40, and >40 kg. Multiple logistic regression analysis examined the association between the subdivided rolling weight and LBP.

**Results:**

No significant differences in odds ratio (OR) of LBP were found for workers handling ≤40 kg rolling weights compared with that for no-handling workers. However, workers handling >40 kg rolling weights had a significantly greater OR of LBP than those not handling loads.

**Conclusions:**

Rolling weights between 40 and 400 kg could place a high stress on the lower back. Implementation in Japan of the HSE recommendations regarding rolling load should be carefully considered.

## Key points:

- Manual rolling of heavy objects such as oil drums, gas cylinders, tires, and rolls of paper and stainless-steel plates occurs at worksites.
- The Health and Safety Executive (HSE) in the United Kingdom recommends rolling weights <400 kg.
- Rolling weights falling within the HSE-recommended 40-400 kg range may increase the risk of low back pain.
- Applying the HSE recommendation for rolling load in Japan should be carefully considered.

## 1. Introduction

Manual rolling of heavy objects such as oil drums, gas cylinders, tires, and rolls of paper and stainless-steel plates still occurs in the contemporary workplace. The rolling task includes a range of activities, such as rolling and moving an empty drum into a washing machine or a rolled stainless-steel plate to a weighing station. The act of rolling objects is classified as pushing,^1^ and these postures have been shown to increase shear forces and compressive forces exerted on the lumbar intervertebral disk.^2^^-^^6^

During rolling, the upper part of a cylindrical object placed on its side is pushed for rotation; the ease of which varies significantly depending on the object’s weight, with heavy objects requiring substantial force.^7^ The Health and Safety Executive (HSE) in the United Kingdom provides a risk assessment of pushing and pulling (RAPP) tool that offers guidance for preventing musculoskeletal disorders in manual material handling.^1^ According to the RAPP tool, the recommended load weight in the rolling task should be <400 kg. In comparison, the weight limits for pushing a wheelbarrow and sliding an object, classified in the same category, are set at 50 and 25 kg, respectively. Thus, the recommended weight for rolling loads notably surpasses those established for pushing and sliding. The force necessary to move a rolling object in motion decreases as the object’s diameter increases.^7^ Rolling loads of <400 kg, as recommended by the HSE, are assumed to be for large-diameter objects. However, this heavier weight can potentially strain the lower back, especially when going over bumps and changing directions. Additionally, the potential for severe injuries exists if the rolling object topples or becomes entangled with a worker.^8^

Consequently, even when adhering to the 400-kg limit for rolling loads, workers may face a risk of work-related low back pain (LBP). However, the association of rolling weights <400 kg with LBP has not been sufficiently investigated. Therefore, this study aimed to examine the effect of rolling loads weighing <400 kg on the occurrence of LBP among workers.

## 2. Methods

### 2.1. Research design

This internet-based cross-sectional survey involved Japanese male and female workers aged 20-75 working in manufacturing, wholesale and retail trade, construction, and transport and postal activities, which entail regular handling of materials and heavy loads. The combined workforce in Japan amounted to 29.4 million individuals in these 4 industries.^9^ The data were gathered from a sample of 30 000 workers, with 7500 workers per industry, based on the sex and age distribution in the labor force survey.^9^

### 2.2. Questionnaire

The survey gathered data on fundamental demographic details, job-related characteristics, job stress factors, working postures, manual handling conditions, regularly managed load weights, and the severity of LBP. These questionnaire items were similar to those used in our previous studies.^10^^,^^11^ Data were extracted from the questionnaire for rolling and no-handling loads. For questioning on manual handling conditions, regularly managed load weights, and the severity of LBP, the initiation of the first LBP episode experienced by a worker in their current job was considered as the reference point for inquiry.

#### 2.2.1. Basic information

Fundamental demographic and job-related characteristics encompassed sex, age, body height and weight, body mass index, smoking status, industry, and the total number of working hours per week.

**Figure 1:**
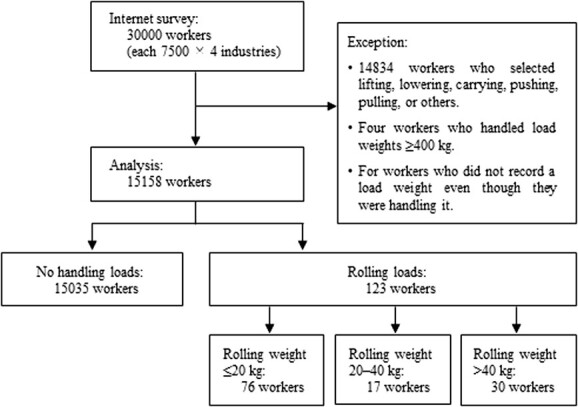
Flow chart showing participation in this study.

**Table 1:** Basic information of workers.

	**No handling (*n* = 15 035)**	**Rolling weight ≤20 kg (*n* = 76)**	**Rolling weight 20-40 kg (*n* = 17)**	**Rolling weight >40 kg (*n* = 30)**	***P* value** ^**a**^
**Age, mean ± SD, y**	47.6 ± 12.7	43.3 ± 10.9	42.5 ± 8.9	43.0 ± 14.8	<.001
**Body height, mean ± SD, cm**	166.2 ± 8.5	168.2 ± 8.8	169.4 ± 10.2	168.4 ± 7.4	.049
**Body weight, mean ± SD, kg**	63.3 ± 13.4	65.0 ± 15.1	68.9 ± 11.6	65.5 ± 12.8	.096
**Body mass index, mean ± SD,**	22.8 ± 3.9	22.8 ± 3.9	24.0 ± 3.4	23.1 ± 4.6	.378
**Sex, *n* (%)**					.043
**Male**	9607 (64)	51 (67)	15 (88)	24 (80)	
**Female**	5428 (36)	25 (33)	2 (12)	6 (20)	
**Smoking status, *n* (%)**					.002
**Nonsmoking**	8464 (56)	35 (46)	4 (24)	9 (30)	
**Smoked in the past**	2849 (19)	20 (26)	7 (41)	8 (27)	
**Smoking**	3722 (25)	21 (28)	6 (35)	13 (43)	
**Industry, *n* (%)**					.163
**Manufacturing**	4034 (27)	19 (25)	3 (18)	7 (23)	
**Wholesale and retail trade**	3639 (24)	15 (20)	3 (18)	7 (23)	
**Construction**	4248 (28)	32 (42)	5 (29)	6 (20)	
**Transportation and postal activities**	3114 (21)	10 (13)	6 (35)	10 (33)	
**Total weekly working hours, *n* (%)**					.744
**<35 h**	2962 (20)	11 (15)	2 (12)	8 (27)	
**35-40 h**	3662 (24)	21 (28)	5 (29)	4 (13)	
**40-45 h**	4357 (29)	20 (26)	4 (24)	7 (23)	
**45-50 h**	1963 (13)	10 (13)	3 (18)	5 (17)	
**≥50 h**	2091 (14)	14 (18)	3 (18)	6 (20)	
**Job demand, *n* (%)**					.085
**Low stress (3-7 points)**	7921 (53)	48 (63)	7 (41)	20 (67)	
**High stress (8-12 points)**	7114 (47)	28 (37)	10 (59)	10 (33)	
**Job control, *n* (%)**					<.001
**Low stress (3-7 points)**	9871 (66)	33 (43)	10 (59)	11 (37)	
**High stress (8-12 points)**	5164 (34)	43 (57)	7 (41)	19 (63)	
**Worksite social support, *n* (%)**					.002
**Low stress (6-15 points)**	7452 (50)	21 (28)	9 (53)	14 (47)	
**High stress (16-24 points)**	7583 (50)	55 (72)	8 (47)	16 (53)	
**Working posture during work, *n* (%)**					.058
**Proper posture**	9667 (64)	55 (72)	14 (82)	17 (57)	
**Forward-bending position**	2296 (15)	9 (12)	1 (6)	3 (10)	
**A half-crouching position**	683 (5)	4 (5)	0 (0)	1 (3)	
**Twisting posture**	845 (6)	4 (5)	0 (0)	4 (13)	
**Unstable posture**	364 (2)	3 (4)	2 (12)	4 (13)	
**Other postures**	236 (2)	0 (0)	0 (0)	0 (0)	
**Multiple above improper postures**	944 (6)	1 (1)	0 (0)	1 (3)	

a The Kruskal-Wallis test for age, body height and weight, and body mass index and the χ^2^ test for the other items were performed to compare 4 weight groups: no-handling plus rolling weights of ≤20, 20-40, and >40 kg.

**Table 2:** Characteristics of the rolling task.

	**Rolling weight ≤20 kg (*n* = 76)**	**Rolling weight 20-40 kg (*n* = 17)**	**Rolling weight >40 kg (*n* = 30)**	***P* value** ^**a**^
**Hours spent in rolling per day, *n* (%)**				<.001
**<1 h**	17 (22)	4 (24)	1 (3)	
**1-2 h**	38 (50)	4 (24)	8 (27)	
**2-3 h**	8 (11)	1 (6)	5 (17)	
**3-4 h**	3 (4)	3 (18)	7 (23)	
**≥4 h**	10 (13)	5 (29)	9 (30)	
**Number of times rolling performed per day, *n* (%)**				.056
**<3 times**	49 (65)	12 (71)	10 (33)	
**3-5 times**	8 (11)	3 (18)	4 (13)	
**5-10 times**	8 (11)	1 (6)	10 (33)	
**10-30 times**	7 (9)	1 (6)	4 (13)	
**≥30 times**	4 (5)	0 (0)	2 (7)	
**Average rolling distance each time, *n* (%)**				<.001
**<1 m**	58 (76)	5 (29)	19 (63)	
**1-5 m**	4 (5)	8 (47)	6 (20)	
**5-10 m**	4 (5)	2 (12)	3 (10)	
**10-20 m**	2 (3)	1 (6)	0 (0)	
**≥20 m**	8 (11)	1 (6)	2 (7)	

a The χ^2^ test was used to compare 3 weight groups: rolling weights of ≤20, 20-40, and >40 kg.

**Table 3:** Association of severe low back pain with rolling weights using multiple logistic regression analyses.

	**Severe LBP, *n* (%)**	**Nonsevere LBP, *n* (%)**	**OR (95%CI)**	***P* value**
**Rolling weight**				
**No handling (0 kg)**	3371 (22)	11 664 (78)	1.00 (Reference)	
**≤20 kg**	17 (22)	59 (78)	1.17 (0.67-2.04)	.582
**20-40 kg**	2 (12)	15 (88)	0.47 (0.11-2.08)	.319
**>40 kg**	16 (53)	14 (47)	3.93 (1.81-8.52)	<.001
**(Adjusted variables)**				
**Sex**				
**Male**	2477 (26)	7220 (74)	1.00 (Reference)	
**Female**	929 (17)	4532 (83)	0.74 (0.68-0.82)	<.001
**Age**				
**20-39 y**	764 (18)	3572 (82)	1.00 (Reference)	
**40-49 y**	881 (23)	2997 (77)	1.50 (1.33–1.68)	<.001
**50-59 y**	931 (25)	2776 (75)	1.81 (1.61–2.03)	<.001
**60-75 y**	830 (26)	2407 (74)	1.95 (1.73–2.21)	<.001
**Body mass index**				
**<18.5**	239 (16)	1262 (84)	1.00 (Reference)	
**≥18.5, <25.0**	2208 (22)	7964 (78)	1.19 (1.02-1.39)	.027
**≥25.0**	959 (28)	2526 (72)	1.45 (1.22-1.72)	<.001
**Smoking status**				
**No smoking**	1585 (19)	6927 (81)	1.00 (Reference)	
**Smoked in the past**	702 (24)	2182 (76)	1.16 (1.04-1.30)	.007
**Smoking**	1119 (30)	2643 (70)	1.46 (1.33-1.61)	<.001
**Industry**				
**Manufacturing**	838 (21)	3225 (79)	1.00 (Reference)	
**Wholesale and retail trade**	754 (21)	2910 (79)	1.05 (0.94-1.19)	.376
**Construction**	995 (23)	3296 (77)	1.03 (0.93-1.15)	.577
**Transportation and postal activities**	819 (26)	2321 (74)	1.16 (1.04-1.31)	.011
**Job demand**				
**Low stress (3-7 points)**	1465 (18)	6531 (82)	1.00 (Reference)	
**High stress (8-12 points)**	1941 (27)	5221 (73)	1.53 (1.41-1.66)	<.001
**Job control**				
**Low stress (3-7 points)**	2083 (21)	7842 (79)	1.00 (Reference)	
**High stress (8-12 points)**	1323 (25)	3910 (75)	1.23 (1.13-1.34)	<.001
**Worksite social support**				
**Low stress (6-15 points)**	1612 (22)	5884 (78)	1.00 (Reference)	
**High stress (16-24 points)**	1794 (23)	5868 (77)	1.08 (0.99-1.17)	.074
**Working posture during work**				
**Proper posture**	1620 (17)	8133 (83)	1.00 (Reference)	
**Forward-bending position**	571 (25)	1738 (75)	1.72 (1.54-1.93)	<.001
**A half-crouching position**	356 (42)	497 (58)	3.56 (3.06-4.14)	<.001
**Twisting posture**	239 (35)	449 (65)	2.76 (2.33-3.28)	<.001
**Unstable posture**	132 (35)	241 (65)	2.41 (1.92-3.02)	<.001
**Other postures**	97 (41)	139 (59)	3.42 (2.60-4.49)	<.001
**Multiple above improper postures**	391 (41)	555 (59)	3.51 (3.03-4.06)	<.001

Abbreviations: LBP, low back pain; OR, odds ratio.

#### 2.2.2. Job stressor

Questions about job stressors were formulated using 3 job demand items (Nos. 1, 2, and 3), 3 job control items (Nos. 8, 9, and 10), and 6 worksite social support items (Nos. 47, 48, 50, 51, 53, and 54) derived from the brief job stress questionnaire.^12^^,^^13^ Participants used a 4-point scale for their responses. Total scores for job demand and control responses fell within the range of 3 to 12. Scores between 3 and 7 points were designated "low stress," whereas scores between 8 and 12 points were designated "high stress." In the case of the 6 worksite social support responses, scores spanned from 6 to 24. Responses garnering 6 to 15 points were designated "low stress," whereas those accumulating 16 to 24 points were designated "high stress."

#### 2.2.3. Working posture

Questions about working posture were evaluated, including proper posture, forward-bending position, a half-crouching position, twisting posture, unstable posture, other postures, and multiple improper postures, allowing multiple answers. Proper posture was defined as maintaining a straight back without exerting excessive force, whereas improper posture was defined as any other than the proper posture.

#### 2.2.4. Load weight

The load weight was specified as the weight value per person involved in regular handling tasks. Load weight was categorized into 4 groups using no-handling loads, the 50th percentile (20 kg), and 75th percentile (40 kg) of the handled weights: no-handling (0 kg) and rolling weights of ≤20 kg, 20-40 kg, and >40 kg (40-400 kg).

#### 2.2.5. Manual handling conditions

Questions regarding manual handling conditions encompassed details such as the handling situation, duration of handling, frequency per day, and the average handling distance. The handling conditions considered were no-handling, rolling, lifting and lowering, carrying, pushing, pulling, and others, allowing multiple answers. This study focused on no-handling and rolling. Participants reported duration of handling in categories of <1, 1-2, 2-3, 3-4, and ≥4 hours. The frequency of handling loads by hand per day was categorized as <3, 3-5, 5-10, 10-30, and ≥30 times. Moreover, participants specified average handing distances as <1, 1-5, 5-10, 10-20, and ≥20 m.

#### 2.2.6. Low back pain

LBP was defined as discomfort felt in the lower back or buttocks, encompassing sensations of pain and numbness extending to the legs, lasting for more than a day. This definition did not include discomfort related to menstruation, pregnancy, or cold conditions. The severity of LBP was categorized into 4 grades using the classification system established by Von Korff et al^14^: grade 0 (no LBP), grade 1 (LBP not interfering with work), grade 2 (LBP interfering with work but without sick leave), and grade 3 (LBP interfering with work and requiring sick leave). Grades 0 and 1 were classified as "non-severe LBP," whereas grades 2 and 3 were classified as "severe LBP."

### 2.3. Survey procedure

Data were gathered through a web-based questionnaire distributed to workers registered with various research-monitoring companies via an internet research company from early to late January 2022. Data collection for each industry was systematically terminated upon reaching 7500 participants.

Participants were provided with information concerning the study’s objective and procedure and assured that their personal information would be treated with strict confidentiality. Informed consent was obtained from each participant before their involvement in the study. This research adhered to the principles outlined in the Declaration of Helsinki and received approval from the ethics board of the National Institute of Occupational Safety and Health of Japan (registration ID: 2021 N29).

### 2.4. Data analysis

#### 2.4.1. Exclusion criteria

Workers engaging in lifting, lowering, carrying, pushing, pulling, or other actions were excluded from the analysis. This exclusion criterion was applied even when rolling was included alongside other activities. Additionally, workers handling loads of ≥400 kg and those who did not provide information on the weight of the loads they handled were excluded from the analysis.

#### 2.4.2. Statistical method

The Kruskal-Wallis test for age, body height and weight, and body mass index and the χ^2^ test for the other items were performed to compare 4 weight groups (no-handling and rolling weights of ≤20, 20-40, and >40 kg) or 3 weight groups (rolling weights of ≤20, 20-40, and >40 kg). For severe LBP, 2 × 2 χ^2^ tests with Bonferroni correction were performed as the post hoc analysis. Multiple logistic regression analysis, incorporating all parameters into the model, was conducted to investigate the association between the categorized weight of rolling loads and LBP. The model computed the odds ratio (OR) and 95% CI. The dependent variable was LBP (severe LBP vs nonsevere LBP) and the independent variable was the categorized weights of rolling loads (rolling weight ≤20, 20-40, or >40 kg vs no-handling). The model was adjusted for sex, age, body mass index, smoking status, industry, job demand and control, worksite social support, and working posture, with all variables treated as categorical data. The variance inflation factor of the independent and adjusted variables was <1.2, indicating no multicollinearity issues. Statistical analyses were conducted using IBM SPSS software version 27, and a significance level of *P* < .05 was considered indicative of statistical significance for tests.

## 3. Results

### 3.1. Target of analysis

Data were analyzed from 15 158 workers, comprising 15 035 workers who did not handle loads and 123 workers who engaged in rolling tasks (Figure 1). Excluded workers included 14 834 who selected lifting, lowering, carrying, pushing, pulling, or other actions, 4 who handled load weights of ≥400 kg, and 4 who did not record a load weight they had handled.

### 3.2. Basic information of workers

Workers performing the rolling task were characterized by being younger and having a higher body height than those who did not handle loads and included a high proportion of males and smokers (Table 1). Rolling task workers also exhibited higher stress regarding job control and worksite social support than no-handling load workers. No significant differences between the no-handling and rolling task workers were observed in body weight, body mass index, industry, total weekly working hours, job demand, or working posture.

### 3.3. Rolling characteristics

Weights handled by rolling task workers ranged from 1 to 350 kg, with an average (±SD) of 39.8 ± 61.3 kg. Among rolling task workers, the weight categories of workers who handled heavier weights spent longer hours in this task (Table 2). Most workers handling weights ≤40 kg reported handling loads less than 3 times per day. Rolling task workers primarily dealt with average load distances of <1 m.

### 3.4. Association of severe LBP with rolling weights

In the no-handling and ≤20, 20-40, and >40 kg rolling weight categories, 22%, 22%, 12%, and 53% of workers had experienced severe LBP, respectively (Table 3). The prevalence of severe LBP among workers handling >40 kg rolling weights was significantly greater than in other weight categories (*P* < .05). The multiple logistic regression analysis revealed no significant differences in the OR of severe LBP for rolling task workers handling ≤20 kg and 20-40 kg compared with that of no-handling workers (Table 3). However, rolling task workers handling >40 kg resulted in a significantly greater OR of severe LBP than those who did not handle loads (OR: 3.93; 95% CI: 1.81-8.52).

## 4. Discussion

The study examined the effect of working with manual rolling weights <400 kg, as recommended by the United Kingdom’s HSE, on the occurrence of LBP among workers. Severe LBP prevalence ranged from 12% to 22% among no-handling workers and the rolling task workers handling ≤20 kg and 20-40 kg, whereas this was significantly higher at 53% among the rolling task workers handling >40 kg. In analyses using the same dataset as this study, 39% of lifting, lowering, and carrying task workers^10^ and 39% of pushing and pulling task workers^11^ complained of severe LBP. Moreover, Fujii and Matsudaira^15^ reported that 38% of Japanese workers, housewives, and unemployed persons aged 20-79 years experienced severe LBP that interfered with work and life. Hence, these results suggest the severe LBP prevalence is higher among workers handling loads >40 kg.

Workers rolling weights ≤20 and 20-40 kg maintained a low OR of LBP similar to those with no-handling loads. The lowest OR of LBP was found in rolling weights of 20-40 kg; however, considering the lack of statistical significance, the OR may change with increased sampling size. In contrast, workers rolling weights >40 kg had a significantly greater OR of LBP. The threshold value of 40 kg was also obtained for the weight category subdivided into increments of 10 kg, even though data availability limited precise calculations. To our knowledge, an association between LBP and rolling loads has not been recently reported. Moreover, the association between LBP and pushing loads, which is in the same category as rolling,^1^ yielded mixed findings.^11^^,^^16^^-^^19^ However, the act of pushing loads was shown to increase shear forces and compressive forces exerted on the lumbar intervertebral disk,^2^^-^^6^ which must pose a risk factor for the development of LBP.

The International Organization for Standardization (ISO) 11 228-2^20^ has established acceptable force thresholds for pushing and pulling activities to mitigate the risk of musculoskeletal disorders but does not provide respective weights for these. The RAPP tool provided by the HSE^1^ recommended weight limits for manual handling activities, including pushing, pulling, sliding, rolling, and others. In this tool, the pushing weight of a wheelbarrow is limited to <50 kg and the sliding weight of an object is limited to <25 kg. Conversely, the recommended weight limit for the rolling task is higher at 400 kg, representing a substantial disparity compared with the limits for pushing and sliding activities.

Rolling resistance is proportional to weight and inversely proportional to the diameter of the wheel or cylinder.^7^ A heavy object is difficult to move because of high rolling resistance. However, with a large object diameter, the rolling resistance reduces, and the object moves more easily. The diameter of rolling objects was not mentioned in the RAPP tool^1^ and was not investigated in this study. The RAPP tool’s recommendation of maintaining weights <400 kg may be predicated on the assumption of larger object diameters, which translates into reduced rolling resistance. However, this premise is no longer appropriate as contemporary workplaces have witnessed mechanization, automation, and systemization advancements,^21^ which contribute to reducing occupational injuries.^22^^,^^23^ Consequently, the dimension of objects used in manual handling in the modern workplace may be smaller than previously presumed for safety. A heavy object weighing several hundred kilograms could cause serious injuries if it falls over or becomes entangled with a worker.^8^ Considering these safety concerns, the rolling weight should be as light as possible. Therefore, although further research is required, including an examination of object diameters, rolling weights of ≤40 kg seem reasonable in contemporary workplaces.

Other than rolling weight, working posture was significantly associated with LBP. Specifically, the adjusted OR was high for a half-crouching position (deep bending posture) and twisting posture. Although these postures are unlikely to be independent causes of LBP,^24^ they increase compressive and shear forces on the lumbar intervertebral disk.^25^ Therefore, maintaining a proper working posture during rolling tasks helps prevent LBP.

The study has several limitations besides the object’s diameter. First, the web-based questionnaire in this study was limited to individuals affiliated with research-monitored companies. This could introduce a degree of sampling bias, as the sample may not fully represent the broader population of workers. However, efforts were made to mitigate the impact of this potential bias by gathering a questionnaire distributed to male and female workers aged 20-75 registered with various research-monitoring companies. Second, this study did not investigate the friction coefficients with the floor. Although this factor could potentially affect the findings, most workplace floors in Japan are maintained with a flat surface. Third, the adjusted variables of multiple logistic regression analysis did not include hours spent rolling, number of rolling times, and average rolling distance because of the insufficient sample size. These factors will influence LBP. In ISO 11228-1,^26^ limit values are established for both weight and cumulative weight (weight × number of times). Although this study focused on weight values, considering cumulative weight is also necessary. Finally, recall bias may influence the results because participants retrospectively reported past work and physical conditions. Further research is warranted to address these limitations.

## 5. Conclusion

The LBP risk in rolling tasks handling ≤40 kg was similar to that of no-handling loads, whereas rolling weights of >40 kg revealed a greater LBP risk than no-handling loads. Rolling weights of >40 kg could place a high stress on the lower back among workers. Consequently, rolling weights falling within the HSE-recommended 40-400 kg range may increase the LBP risk. Moreover, applying the HSE recommendation for rolling loads in Japan should be carefully considered. Future studies should examine the effects of reducing LBP risk with manual rolling loads of ≤40 kg.
